# In Vitro Effects of an Olive Polyphenol–Bromelain Formulation on U87MG Glioblastoma Cells: Viability, Cell-Cycle Distribution, Cytokine Release, and Total Antioxidant Capacity

**DOI:** 10.3390/molecules31183334

**Published:** 2026-09-20

**Authors:** Enver Ciraci, Tugba Elgun, Yasemin Musteri Oltulu, Halil Ibrahim Arslan, Asiye Gok Yurttas, Gul Ipek Gundogan, Lokman Uzun, Gurol Emekdas

**Affiliations:** 1 Department of Biochemistry, Faculty of Pharmacy, Biruni University, Zeytinburnu, 34010 Istanbul, Türkiye; eciraci@biruni.edu.tr; 2 Biruni Advanced Technology Application and Research Center (BAMER), Biruni University, 34015 Istanbul, Türkiye; telgun@biruni.edu.tr (T.E.); yoltulu@biruni.edu.tr (Y.M.O.); halila@biruni.edu.tr (H.I.A.); ggundogan@biruni.edu.tr (G.I.G.); 3 Department of Medical Biology, Faculty of Medicine, Biruni University, Zeytinburnu, 34015 Istanbul, Türkiye; 4 Department of Biochemistry, Faculty of Medicine, Istanbul Atlas University, 34408 Istanbul, Türkiye; 5 Department of Histology and Embryology, Faculty of Medicine, Biruni University, 34015 Istanbul, Türkiye; 6 Department of Chemistry, Faculty of Science, Hacettepe University, 06800 Ankara, Türkiye; lokman@hacettepe.edu.tr; 7 Turk Kan Vakfi, 34718 Istanbul, Türkiye; gemekdas@gmail.com

**Keywords:** glioblastoma, U87MG, olive polyphenols, bromelain, cell-cycle distribution, G0/G1 accumulation, IL-6, IL-10, total antioxidant capacity

## Abstract

**Background:** Glioblastoma (GBM) remains difficult to treat, and natural bioactive formulations are being explored as experimental modifiers of tumor-associated cellular processes. This study examined a fixed-ratio olive polyphenol–bromelain formulation in U87MG cells and performed targeted phenolic characterization of its olive-derived component. **Methods:** Seven phenolics were quantified by LC–MS/MS. U87MG cells were exposed to the formulation or a bromelain comparator. The MTT metabolic signal, DNA-content cell-cycle distribution, IL-6, IL-10, and extracellular total antioxidant capacity (TAC) were assessed. **Results:** The total quantified phenolics were 483.22 mg/kg, with oleocanthal, oleuropein, and 3,4-DHPEA-EDA predominating. The formulation reduced the MTT signal more than bromelain at lower and intermediate concentrations, although the response became non-monotonic at higher concentrations. Combination-treated cells showed greater G0/G1 accumulation. Culture supernatants showed lower IL-6 and higher IL-10 and TAC relative to untreated controls. **Conclusions:** The formulation was associated with changes in metabolic activity, cell-cycle distribution, and extracellular cytokine/redox readouts in U87MG cells. Because no polyphenol-only arm, direct cell-death assay, complete replicate-level inferential analysis, or non-tumoral comparator was available, the findings are descriptive and do not establish synergy, cytotoxicity, mechanism, tumor selectivity, or therapeutic efficacy.

## 1. Introduction

Glioblastoma (GBM) is the most aggressive adult-type diffuse glioma and is currently defined within the integrated histomolecular framework of the World Health Organization classification as glioblastoma, IDH-wildtype, CNS WHO grade 4. This classification emphasizes that molecular features are essential for accurate diagnosis and for distinguishing GBM from IDH-mutant astrocytic tumors with different biological behavior. Clinically, GBM is characterized by rapid proliferation, diffuse infiltration into surrounding brain tissue, extensive intratumoral heterogeneity, hypoxia, necrosis, angiogenesis, and profound resistance to therapy [1,2].

Despite maximal safe surgical resection followed by radiotherapy with concurrent and adjuvant temozolomide, and in selected settings tumor-treating fields, patient outcomes remain poor. Recent clinical reviews and consensus statements continue to report median overall survival mostly within the range of 12–18 months, with recurrence being almost inevitable in most patients [2,3,4,5]. These limitations have increased interest in adjunctive strategies that can target tumor-cell proliferation, inflammatory signaling, oxidative stress, and therapy-resistant cellular states without adding substantial toxicity.

Natural bioactive compounds have attracted attention because many of them act on multiple cancer-relevant pathways simultaneously rather than on a single molecular target. Polyphenols are among the best studied natural compounds in this context. Olive-derived polyphenols, particularly hydroxytyrosol, oleuropein and related phenolic secoiridoids, have been associated with antioxidant, anti-inflammatory, anti-proliferative and pro-apoptotic effects in several experimental cancer models [6,7]. Recent reviews specifically highlight the potential relevance of hydroxytyrosol in brain tumors because of its capacity to influence oxidative stress, mitochondrial function, inflammatory mediators, cell survival pathways and, importantly, its potential to reach the central nervous system [6].

Mechanistically, polyphenols may interfere with glioblastoma biology by regulating NF-kB, PI3K/AKT/mTOR, MAPK, STAT3 and p53/p21-related pathways, which are closely linked to proliferation, cell-cycle progression, apoptosis, invasion and inflammatory microenvironmental remodeling [6,7,8,9]. Several polyphenols, including resveratrol, curcumin, quercetin and epigallocatechin gallate, have been shown to induce cell-cycle arrest and cell death in glioma models, although their bioavailability and delivery remain translational challenges [8,9,10,11,12]. Olive polyphenols may therefore be considered part of a broader class of nutraceutical-derived agents with potential adjunctive value in GBM research.

Bromelain is a complex mixture of cysteine proteases mainly obtained from pineapple (Ananas comosus). It has been studied for its anti-inflammatory, immunomodulatory, wound-healing and anticancer properties [13,14,15]. Recent reviews describe bromelain as a multifunctional bioactive compound capable of modulating apoptosis, angiogenesis, inflammatory pathways, immune responses and cell adhesion processes [13,14]. In cancer models, bromelain has been reported to affect NF-kB signaling, reactive oxygen species generation, autophagy, apoptosis and cell-cycle progression [15,16]. In glioma models specifically, bromelain has been shown to reversibly reduce invasive properties of glioma cells, partly through proteolytic effects on cell-surface adhesion molecules such as CD44 and integrins [17].

The rationale for combining olive-derived polyphenols with bromelain is their potentially complementary biological activity. Polyphenols can influence redox balance, inflammatory signaling, and cell-cycle regulation, whereas bromelain may affect protease-sensitive membrane proteins and inflammatory mediators. A combination could therefore yield a response distinct from either constituent alone. However, an enhanced effect cannot be termed additive or synergistic without testing both components individually across an appropriate dose matrix and applying a formal interaction model. Evidence specifically addressing polyphenol–bromelain combinations in glioblastoma remains limited.

Inflammatory cytokines are particularly relevant in GBM. IL-6 is a major tumor-promoting cytokine that can activate JAK/STAT3 signaling, support glioma stem-like cell maintenance, promote immune suppression, and contribute to treatment resistance [18,19,20]. IL-10 has context-dependent roles in the tumor microenvironment and should not be interpreted as a uniformly favorable anti-inflammatory marker. In a tumor-cell monoculture, changes in IL-10 are therefore best considered alongside other cytokine readouts rather than as evidence of a beneficial immune effect [21,22].

The present study therefore evaluated an olive polyphenol–bromelain formulation in U87MG human glioblastoma cells. The prespecified experimental readouts were metabolic viability, DNA-content cell-cycle distribution, IL-6 and IL-10 concentrations in culture supernatants, and total antioxidant capacity. The study was designed as an exploratory comparison of the combination formulation with bromelain alone and untreated cells; it was not designed to establish pharmacological synergy or a specific molecular mechanism.

## 2. Results

### 2.1. Targeted Phenolic Characterization of the Olive Oil Component

Targeted LC–MS/MS quantification identified seven phenolic constituents in the olive oil component, with a cumulative concentration of 483.22 mg/kg. Oleocanthal (p-HPEA-EDA) was the most abundant quantified compound (132.58 mg/kg), followed by oleuropein (124.16 mg/kg) and 3,4-DHPEA-EDA (96.41 mg/kg). Hydroxytyrosol (42.27 mg/kg), oleuropein aglycon (38.91 mg/kg), ligstroside aglycon (30.76 mg/kg), and tyrosol (18.13 mg/kg) were also quantified (Table 1).

### 2.2. MTT Metabolic Viability Signal in U87MG Cells

**Figure 1:**
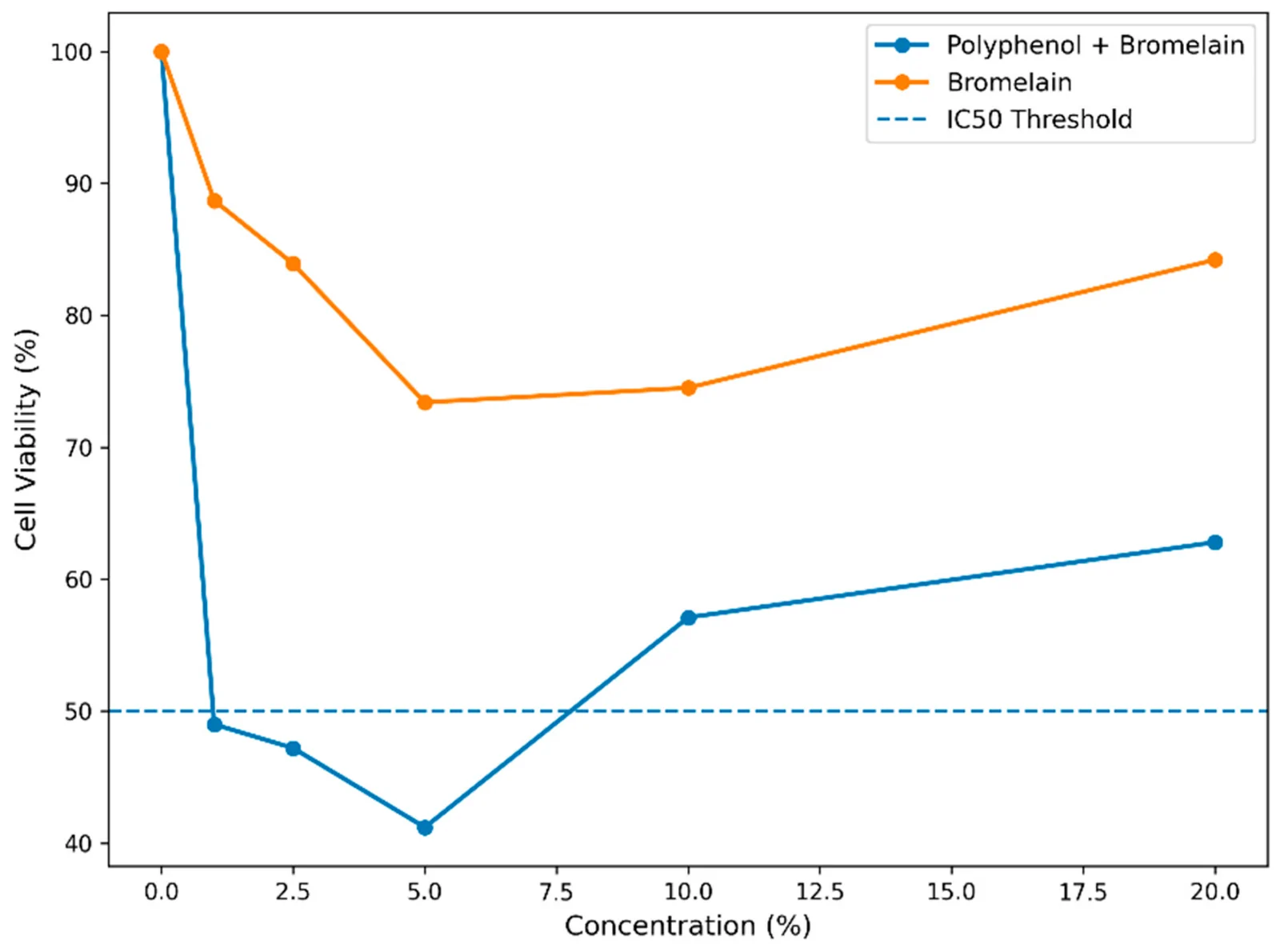
Normalized U87MG MTT metabolic signal after exposure to the fixed-ratio olive polyphenol–bromelain formulation or bromelain alone. The *x*-axis shows nominal formulation concentration, and the dashed line indicates 50% of the untreated-control signal. The formulation response became non-monotonic at the higher concentrations; therefore, no conventional IC50 or inferential significance is assigned to this curve.

The formulation produced a marked reduction in the U87MG MTT signal at the lower and intermediate tested concentrations compared with bromelain alone (Figure 1). The first observed crossing of approximately 50% of the untreated-control signal occurred at the 1% formulation condition, corresponding to approximately 800 µg/mL bromelain and 96 µg/mL olive-derived polyphenols. The lowest formulation-associated MTT signal was observed at 5%, after which the signal increased at 10% and 20%. Bromelain alone did not cross the 50% threshold within the tested range. Because the formulation response was non-monotonic, these data are not treated as a conventional IC50 curve.

### 2.3. Cell-Cycle Distribution

**Figure 2:**
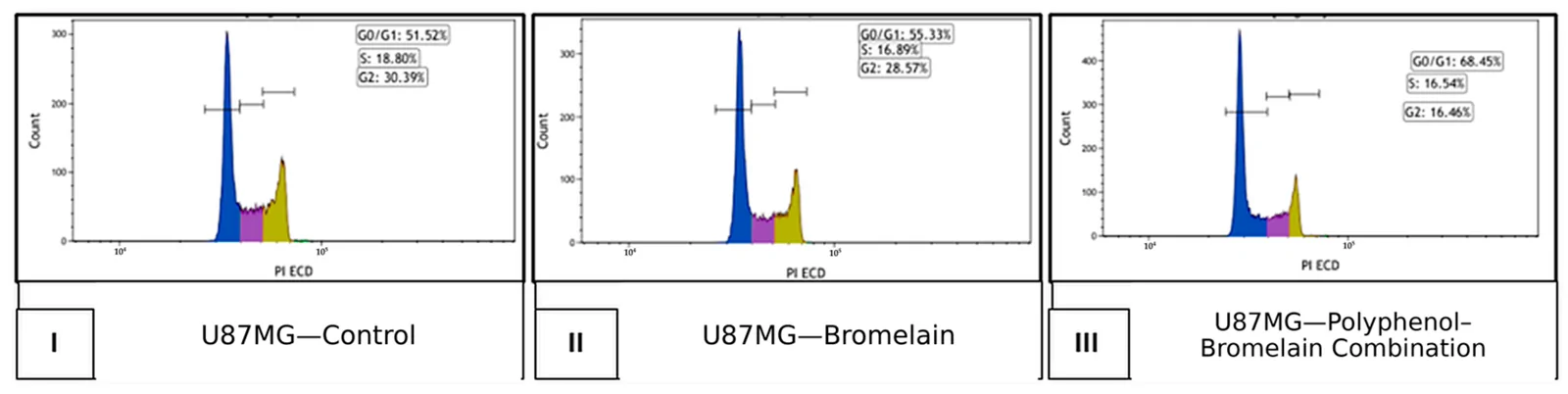
Representative DNA-content flow-cytometric profiles of U87MG cells under untreated-control, bromelain, and olive polyphenol–bromelain formulation conditions. The profiles illustrate the observed distribution of events across G0/G1, S, and G2/M phases. The figure is descriptive; no checkpoint mechanism or cell-death endpoint was directly measured. Blue indicates G0/G1, purple indicates S phase, and yellow indicates G2/M.

Representative DNA-content flow-cytometric profiles differed among untreated, bromelain-treated, and formulation-treated U87MG cells (Figure 2). The formulation-treated profile showed a greater proportion of events in the G0/G1 compartment with a corresponding reduction in actively cycling fractions relative to the untreated profile. This pattern is described as G0/G1 accumulation; it is not interpreted as evidence of a specific checkpoint mechanism or of cell death.

### 2.4. IL-6, IL-10, and Total Antioxidant Capacity in Culture Supernatants

**Figure 3:**
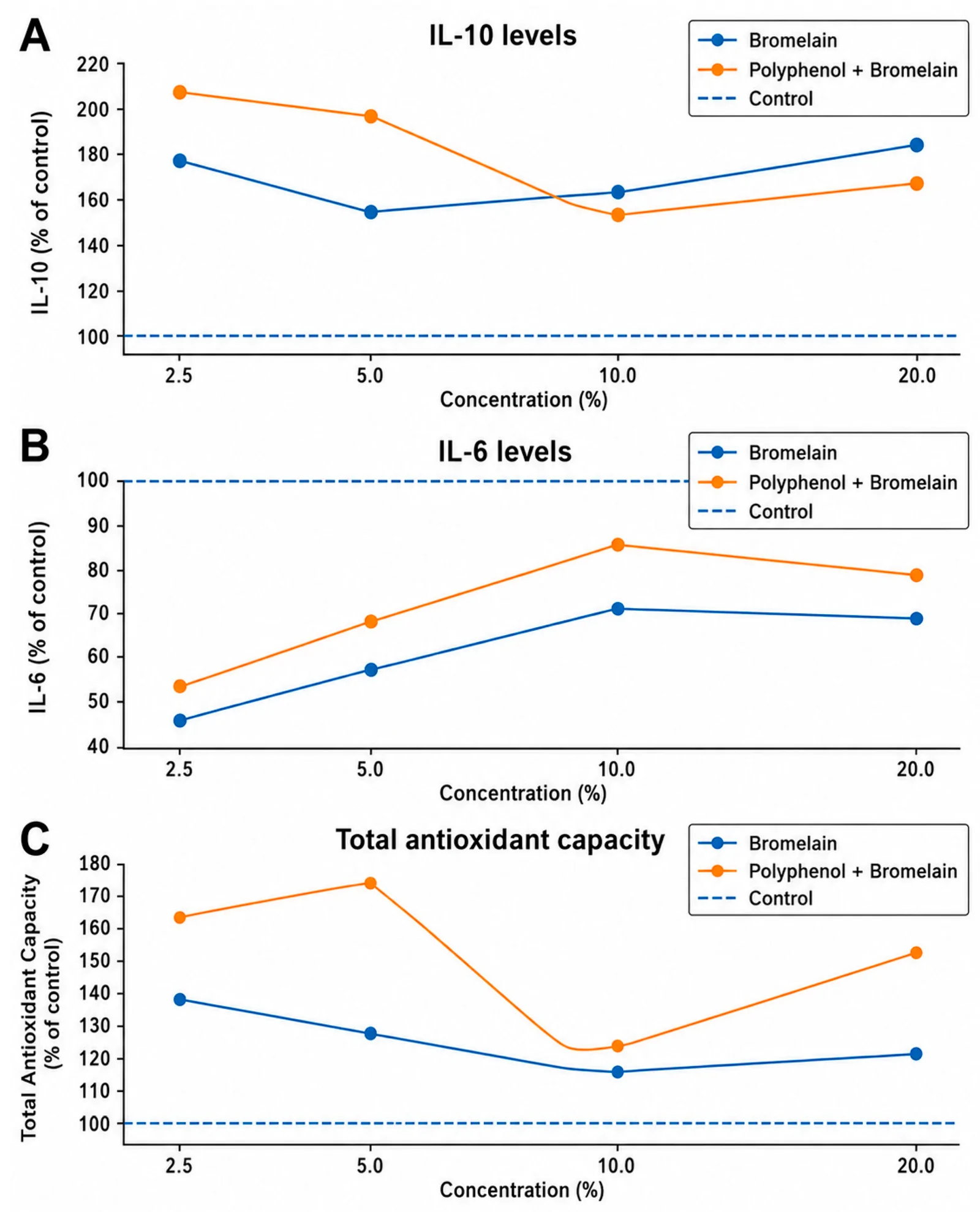
Relative IL-10 (**A**), IL-6 (**B**), and total antioxidant capacity (TAC) (**C**) in U87MG culture supernatants after bromelain or olive polyphenol–bromelain formulation exposure. Values are normalized to untreated control (100%) and are presented as descriptive summary values; no inferential significance is assigned.

IL-6, IL-10, and TAC were evaluated in culture supernatants after bromelain or formulation exposure (Figure 3). Relative to untreated controls, IL-6 values were lower after both treatments across the reported concentration range, whereas IL-10 values were higher. TAC values were also above the untreated-control level, with the formulation showing higher values than bromelain alone at several tested concentrations. These are descriptive, normalized extracellular readouts; inferential significance is not assigned.

## 3. Discussion

The present data show a coordinated change across several readouts rather than a defined antitumor mechanism. Relative to the bromelain comparator, the fixed-ratio formulation produced a larger reduction in the MTT signal at lower and intermediate concentrations, a representative shift toward G0/G1 accumulation, and changes in extracellular IL-6, IL-10, and TAC. These findings are informative at the level of an exploratory U87MG model, but the design does not support claims of pharmacological synergy, direct cytotoxicity, tumor selectivity, or therapeutic efficacy.

Targeted LC–MS/MS established that oleocanthal, oleuropein, and 3,4-DHPEA-EDA were the predominant quantified phenolics, with hydroxytyrosol, oleuropein aglycon, ligstroside aglycon, and tyrosol also present. This improves the definition of the olive-derived component, but it remains a targeted seven-analyte profile rather than a comprehensive chemical characterization. Unmeasured phenolics and other matrix constituents may also contribute to the observed cellular responses, and the present design cannot attribute an effect to any single compound [6,7,23].

The non-monotonic MTT pattern is particularly important for interpretation. The formulation signal fell below approximately 50% of control at the lower/intermediate concentrations but rose again at 10% and 20%. This should not be taken as evidence that higher concentrations restored proliferation. Tetrazolium assays report metabolic reduction rather than cell number directly, and redox-active polyphenols can alter MTT readouts through metabolic, chemical, or optical interference. Such limitations have been demonstrated with polyphenols generally and specifically in glioma-cell experiments [24,25]. Because no orthogonal viability assay or cell-free formulation control was available, the present study cannot distinguish a biological non-monotonic response from assay-related interference. For this reason, the MTT data are reported as a normalized metabolic signal and not as a definitive cytotoxicity curve or formal IC50.

The DNA-content profiles provide a complementary observation. G0/G1 accumulation can accompany reduced cell-cycle progression, and related polyphenol effects have been linked in other models to cyclin/CDK control, p21/p27, PI3K/AKT, NF-κB, and STAT3 signaling [8,9,10,11,12]. None of these checkpoint pathways were measured here. Moreover, DNA-content distribution does not distinguish cytostasis from apoptosis or necrosis. The flow-cytometric result therefore supports a change in cell-cycle distribution but not a specific G1/S checkpoint mechanism or a cell-death pathway.

Bromelain may influence tumor-cell behavior through several routes, including inflammatory signaling, apoptosis-related pathways, and proteolytic effects on adhesion-associated surface molecules [13,14,15,16,17,26]. However, the present comparison includes bromelain alone and the fixed-ratio formulation, but not a matched polyphenol-only arm. The larger response of the formulation therefore cannot be assigned specifically to the olive-derived component, nor can it establish an interaction between the two components. Demonstration of additivity or synergy would require component-specific dose–response curves and a formal interaction design such as Chou–Talalay, Bliss independence, highest-single-agent, or related response-additivity approaches [27,28].

The cytokine findings also require a restrained interpretation. Lower extracellular IL-6 is directionally relevant because IL-6/JAK/STAT3 signaling supports several tumor-promoting features of GBM [18,19,20]. Nevertheless, supernatant IL-6 was not normalized to viable cell number or total cellular protein, so lower values may reflect altered secretion, reduced cell abundance/metabolic activity, or both. IL-10 is more context-dependent and may participate in immunosuppressive signaling within the GBM microenvironment [21,22]. In a U87MG monoculture without microglia, macrophages, lymphocytes, endothelial cells, or astrocytes, an increase in IL-10 cannot be classified as a favorable immunomodulatory effect.

The TAC result should be interpreted with similar caution. TAC was measured in a culture supernatant into which a polyphenol-containing formulation had been added. Consequently, the higher extracellular TAC may reflect, at least in part, the intrinsic reducing capacity of formulation constituents rather than a cell-mediated increase in antioxidant defense. The assay does not establish the direction of intracellular oxidative stress. Cell-free formulation controls together with intracellular ROS, glutathione, lipid-peroxidation, mitochondrial, or oxidative-DNA-damage measurements would be needed to resolve that question.

Taken together, the value of the present study lies in linking a defined targeted phenolic profile with several cellular and extracellular observations in the same experimental model. The findings generate testable hypotheses but do not identify the responsible component or molecular pathway. Replication in a molecularly distinct GBM model, a non-tumoral neural comparator, and patient-derived or three-dimensional systems would be required to determine reproducibility and biological selectivity [29,30].

### Study Limitations

Several limitations are central to interpretation. The study used a single established U87MG cell line and did not include a polyphenol-only arm, a non-tumoral neural comparator, or direct apoptosis/necrosis measurements. The targeted chemical panel quantified seven phenolics but did not provide untargeted compositional coverage. The MTT assay was not confirmed by an orthogonal viability method, and the non-monotonic high-concentration response may therefore include assay-related effects. In addition, complete replicate-level numerical data were not available for retrospective inferential analysis, so the biological results are presented descriptively.

IL-6, IL-10, and TAC were measured in supernatants without normalization to viable cell number or total cellular protein. TAC may also include the direct reducing contribution of the polyphenol-containing formulation. No pathway-level measurements, patient-derived model, three-dimensional system, or in vivo validation were included. These limitations preclude conclusions regarding cell-death mechanism, pharmacological synergy, tumor selectivity, normal-cell safety, bioavailability, blood–brain barrier penetration, or therapeutic efficacy.

## 4. Materials and Methods

### 4.1. Targeted LC–MS/MS Characterization of the Olive Oil Component

The Gemlik olive oil component used in the formulation was subjected to targeted phenolic analysis by liquid chromatography–tandem mass spectrometry (LC–MS/MS) as an external analytical service at the Natural Products Application and Research Center, Süleyman Demirel University (Isparta, Türkiye). Seven phenolic constituents were quantified and reported as mg/kg of the analyzed olive oil sample. The archived analytical report specified an analytical reporting limit of 0.05 mg/kg. The analysis is therefore described here as targeted phenolic characterization rather than comprehensive untargeted compositional profiling.

### 4.2. Cell Culture and Treatment Design

The human glioblastoma cell line U87MG (ATCC HTB-14; RRID: CVCL_0022) was used as the experimental model. Cells were cultured in Dulbecco’s Modified Eagle Medium (DMEM; Gibco, Thermo Fisher Scientific, Waltham, MA, USA) supplemented with 10% fetal bovine serum (FBS), 1% penicillin–streptomycin, and 1% L-glutamine. Cultures were maintained at 37 °C in a humidified incubator containing 5% CO2. The medium was renewed every two days, and cultures were routinely monitored for morphology and contamination.

For treatment, U87MG cells were seeded into 96-well plates at 1 × 10^5^ cells per well and allowed to adhere for 24 h. Cells were then exposed to the olive-derived polyphenol–bromelain formulation (SammyVit, Balgat, Ankara, Türkiye) at nominal final formulation levels of 1%, 2.5%, 5%, 10%, and 20%. These percentages refer to dilution levels of the same fixed-ratio formulation in culture medium and not to the percentage of polyphenol relative to bromelain. Based on the formulation composition used in the experiment, the bromelain:olive-polyphenol mass ratio was 25:3. The 1%, 2.5%, 5%, 10%, and 20% conditions corresponded to approximately 800/96, 2000/240, 4000/480, 8000/960, and 16,000/1920 µg/mL bromelain/polyphenols, respectively. Thus, the component ratio remained constant while the total formulation amount increased. The bromelain-only treatment was tested at the matched nominal concentration points, and untreated cells served as controls. A polyphenol-only arm was not included.

### 4.3. MTT Metabolic Viability Assay

Cellular metabolic reduction capacity was assessed using the MTT assay (Abcam, Cambridge, UK). After treatment, 20 µL of MTT solution (5 mg/mL) was added to each well and incubated for 4 h at 37 °C. Formazan crystals were dissolved in dimethyl sulfoxide (DMSO), and absorbance was measured at 590 nm. The MTT signal was normalized to untreated controls. Because the formulation response became non-monotonic at higher concentrations, a formal pharmacological IC50 was not calculated; only the observed crossing of approximately 50% of the untreated-control MTT signal is reported descriptively.

### 4.4. Cell-Cycle Analysis

Cell-cycle distribution was analyzed by DNA-content flow cytometry using the NutriCulture Cell Cycle Analysis Kit (Ecotech, Istanbul, Türkiye). After treatment, cells were harvested, fixed, and stained according to the manufacturer’s protocol. DNA content was acquired using a BD Accuri C6 flow cytometer (BD Biosciences, Franklin Lakes, NJ, USA), and the proportions of events in G0/G1, S, and G2/M phases were evaluated with FlowJo software (v10.8).

### 4.5. Cytokine and Total Antioxidant Capacity Analyses

Culture supernatants were collected after 48 h of treatment. IL-6 and IL-10 were measured using commercial ELISA kits (BT-LAB, Shanghai Korain Biotech, Shanghai, China), with absorbance recorded at 450 nm and concentrations derived from the corresponding standard curves. Total antioxidant capacity (TAC) was determined in the culture supernatants using a commercial assay according to the manufacturer’s instructions. For presentation, IL-6, IL-10, and TAC values were normalized to untreated controls and expressed as percentages of control.

### 4.6. Statistical Analysis

The assays were conducted in three independent experimental runs. Complete replicate-level numerical values for all endpoints were not available in the archived dataset used for the present revision. Accordingly, no retrospective inferential statistics were calculated and no *p* values were reconstructed from the figures; the results are presented as normalized descriptive summaries.

## 5. Conclusions

In U87MG cells, the fixed-ratio olive polyphenol–bromelain formulation was associated with a larger reduction in MTT metabolic signal than bromelain alone at lower and intermediate concentrations, G0/G1 accumulation, lower extracellular IL-6, higher IL-10, and higher extracellular TAC. The MTT response became non-monotonic at higher formulation concentrations.

These findings are descriptive and hypothesis-generating. In the absence of a polyphenol-only arm, direct cell-death analysis, orthogonal viability testing, complete replicate-level inferential analysis, and non-tumoral or additional GBM models, the data do not establish pharmacological synergy, cytotoxicity, a specific mechanism, tumor selectivity, or therapeutic efficacy.

## Figures and Tables

**Table 1:** Targeted LC–MS/MS quantification of phenolic constituents in the Gemlik olive oil component used in the polyphenol–bromelain formulation.

Phenolic Constituent	Common/Alternative Designation	Concentration (mg/kg)	Relative Contribution to Quantified Phenolics (%)
p-HPEA-EDA	Oleocanthal	132.58	27.44
Oleuropein	Oleuropein	124.16	25.69
3,4-DHPEA-EDA	Oleuropein-derived secoiridoid	96.41	19.95
3,4-DHPEA	Hydroxytyrosol	42.27	8.75
3,4-DHPEA-EA	Oleuropein aglycon	38.91	8.05
p-HPEA-EA	Ligstroside aglycon	30.76	6.37
p-HPEA	Tyrosol	18.13	3.75
Total		483.22	100.00

Phenolic constituents are expressed as mg/kg of the analyzed olive oil sample. The analytical report specified a reporting limit of 0.05 mg/kg. Relative contribution (%) was calculated from the cumulative concentration of the seven quantified phenolics (483.22 mg/kg).

## Data Availability

The summary data supporting the findings are contained within the article and its figures. Complete replicate-level numerical records for all assays are not available for retrospective re-analysis or public deposition.
